# Influenza-associated Hospitalizations by Industry, 2009–10 Influenza Season, United States

**DOI:** 10.3201/eid1804.110337

**Published:** 2012-04

**Authors:** Sara E. Luckhaupt, Marie Haring Sweeney, Renee Funk, Geoffrey M. Calvert, Mackenzie Nowell, Tiffany D’Mello, Arthur Reingold, James Meek, Kimberly Yousey-Hindes, Kathryn E. Arnold, Patricia Ryan, Ruth Lynfield, Craig Morin, Joan Baumbach, Shelley Zansky, Nancy M. Bennett, Ann Thomas, William Schaffner, Timothy Jones

**Affiliations:** Centers for Disease Control and Prevention, Cincinnati, Ohio, USA (S.E. Luckhaupt, M. H. Sweeney, G.M. Calvert);; Centers for Disease Control and Prevention, Atlanta, Georgia, USA (R. Funk, M. Nowell, T. D’Mello);; University of California, Berkeley, California, USA (A. Reingold);; Yale University, New Haven, Connecticut, USA (J. Meek, K. Yousey-Hindes);; Georgia Division of Public Health, Atlanta (K. E. Arnold);; Maryland Department of Health and Mental Hygiene, Baltimore, Maryland, USA (P. Ryan),; Minnesota Department of Health, Minneapolis, Minnesota, USA (R. Lynfield, C. Morin);; New Mexico Department of Health, Santa Fe, New Mexico, USA (J. Baumbach);; New York State Department of Health, Albany, New York, USA (S. Zansky);; University of Rochester, Rochester, New York, USA (N. M. Bennett);; Oregon Public Health Division, Portland, Oregon, USA (A. Thomas);; Vanderbilt University, Nashville, Tennessee, USA (W. Schaffner);; Tennessee Department of Health, Nashville (T. Jones)

**Keywords:** influenza, pandemic (H1N1) 2009, viruses, hospitalizations, humans, occupational groups, health personnel, industry

## Abstract

Certain industries were overrepresented among employed adults with this disease.

## Medscape CME Activity

Medscape, LLC is pleased to provide online continuing medical education (CME) for this journal article, allowing clinicians the opportunity to earn CME credit. 

This activity has been planned and implemented in accordance with the Essential Areas and policies of the Accreditation Council for Continuing Medical Education through the joint sponsorship of Medscape, LLC and Emerging Infectious Diseases. Medscape, LLC is accredited by the ACCME to provide continuing medical education for physicians.

Medscape, LLC designates this Journal-based CME activity for a maximum of 1 *AMA PRA Category 1 Credit(s)*^TM^. Physicians should claim only the credit commensurate with the extent of their participation in the activity.

All other clinicians completing this activity will be issued a certificate of participation. To participate in this journal CME activity: (1) review the learning objectives and author disclosures; (2) study the education content; (3) take the post-test with a 70% minimum passing score and complete the evaluation at www.medscape.org/journal/eid; (4) view/print certificate.

**Release date: March 14, 2012; Expiration date: March 14, 2013**

## Learning Objectives

Upon completion of this activity, participants will be able to:

- Distinguish the effect of employment on the risk for hospitalization for influenza
- Assess the risk for hospitalization for influenza across different sectors of industry
- Evaluate the interaction between underlying medical illness and the risk for hospitalization for influenza among workers
- Analyze other factors potentially associated with the risk for hospitalization for influenza among workers.

## CME Editor

***Thomas J. Gryczan, ***MS, Technical Writer/Editor,* Emerging Infectious Diseases. Disclosure: Thomas J. Gryczan, MS, has disclosed no relevant financial relationships.*

## CME Author

***Charles P. Vega, MD, ***Health Sciences Clinical Professor; Residency Director, Department of Family Medicine, University of California, Irvine.* Disclosure: Charles P. Vega, MD, has disclosed no relevant financial relationships.*

## Authors

*Disclosures:* ***Sara E. Luckhaupt, MD, MPH; Mackenzie Nowell, MPH; Tiffany D’Mello, MPH; Arthur Reingold, MD; James Meek, MPH; Kimberly Yousey-Hindes; Renee Funk, DVM; Patricia Ryan, MS; Ruth Lynfield, MD; Craig Morin, MPH; Joan Baumbach, MD, MPH; Shelley Zansky, PhD; Nancy M. Bennett, MD, MS; Ann Thomas, MD, MPH;*** *and* ***Timothy Jones, MD*** *, have disclosed no relevant financial relationships.* ***Marie Haring Sweeney, PhD, MPH*** *, has disclosed the following relevant financial relationships: owns stock, stock options, or bonds from P&G, Apple, Lucent, J&J.* ***Geoffrey M. Calvert, MD, MPH*** *, has disclosed the following relevant financial relationships: owns stock, stock options, or bonds from Bristol-Myers Squibb, Abbott, Amgen, Baxter, Gen-Probe, Johnson & Johnson, Pfizer, Gilead, St. Jude Medical, Stryker, Teva, Novartis.* ***Kathryn E. Arnold, MD*** *, has disclosed the following relevant financial relationships: owns stock, stock options, or bonds from Steris Corp, Kimberley Clark Corp.* ***William Schaffner, MD*** *, has disclosed the following relevant financial relationships: served as an advisor or consultant for Merck, sanofi-Pasteur, Pfizer, GSK, Dynavax.*
