# Supplementary material for: Influenza-associated Hospitalizations by Industry, 2009–10 Influenza Season, United States
Source: Emerg Infect Dis. 2012 Apr;18(4):556–62. doi: 10.3201/eid1804.110337 (PMC3309704; doi:10.3201/eid1804.110337)
Supplement: Related texts

# Research article

**DOI:** 10.3201/eid1804.110337

## Abstract

In response to pandemic (H1N1) 2009, data were collected on work status and industry of employment of 3,365 adults hospitalized with laboratory-confirmed influenza during the 2009–10 influenza season in the United States. The proportion of workers hospitalized for influenza was lower than their proportion in the general population, reflecting underlying protective characteristics of workers compared with nonworkers. The most commonly represented sectors were transportation and warehousing; administrative and support and waste management and remediation services; health care; and accommodation and food service.

Although the Occupational Safety and Health Administration, Department of Labor, and the National Institute for Occupational Safety and Health (NIOSH), Centers for Disease Control and Prevention (CDC), have issued guidance to assist workplaces in responding to influenza pandemics (*1**,**2*), more information is needed about which specific groups of workers are at highest risk for acquiring or having complications from pandemic and seasonal influenza. Specifically, this information is needed for recognizing and responding to increased risks for infection among key occupational groups (e.g., health care workers, school teachers, retail and food service workers, and others with substantial exposure to the general public); informing persons who develop guidance for key policy questions, including the prioritization of groups to receive vaccine, school closing policies, and appropriate personal protective equipment use; and providing data that might trigger more in-depth case studies of clusters of disease occurring among specific workers.

During the influenza (H1N1) 2009 pandemic, NIOSH explored multiple sources of data on the occupations of affected persons. The occupational distribution of all confirmed (H1N1) 2009 pandemic influenza case-patients from 4 states during the early phase of the pandemic (April–July 2009) has been reported by Suarthana et al. (*3*) but as the pandemic progressed and case counts rapidly increased, it became impossible to collect occupational information on all case-patients.

Thus, during the fall wave of the pandemic, NIOSH worked with the CDC Emerging Infections Program (EIP) to collect data on the industry and occupation of the subset of adults hospitalized with laboratory-confirmed influenza. Hospitalized case-patients, many of whom have underlying medical risk factors for severe disease, are not representative of all persons who acquire influenza. Thus, studying them provides little insight into the risk of acquiring influenza. However, examining the distribution of industry of employment of these persons provides some clues about specific groups of workers that might be most commonly affected by severe influenza.

## Methods

### EIP Data for Hospitalized Influenza Case-Patients

The EIP is coordinated and funded by CDC. It consists of a network of 10 state health departments (California, Colorado, Connecticut, Georgia, Maryland, Minnesota, New Mexico, New York, Oregon, and Tennessee) and their collaborators in local health departments, academic institutions, other federal agencies, and public health and clinical laboratories. The network comprises a catchment area of ≈16.8 million persons ≥18 years of age. The population studied by the EIP is roughly representative of the US population on the basis of demographic characteristics such as age, sex, race, urban residence, and population density and percentage at or below the poverty level. During the 2009–10 influenza season (September 1, 2009−April 30, 2010), the 10 EIP sites performed active population-based surveillance for laboratory-confirmed influenza-related hospitalizations (see www.cdc.gov/ncpdcid/deiss/eip/index.html for information about EIP). This period coincided with circulation and dominance of the pandemic (H1N1) 2009 virus strain. Institutional Review Board approval for EIP adult influenza-associated hospitalization surveillance activities during the 2009–10 influenza season was obtained from all sites, including CDC.

As described elsewhere (*4*), cases were defined as persons >18 years of age hospitalized for community-onset, laboratory-positive influenza infection. Case-patients were residents of the defined EIP catchment areas and were admitted to a surveillance-area hospital during the influenza season and within 14 days of receiving a positive influenza test result. Laboratory confirmation of influenza was obtained by virus culture, immunofluorescence antibody staining, reverse transcription PCR, or a commercially available rapid diagnostic test. Written documentation of a positive influenza test result in the medical chart was acceptable as evidence of laboratory confirmation. Persons who had positive results for influenza >3 days after hospital admission were considered to have nosocomial influenza and were excluded from this study. Staff at each EIP site identified cases by contacting hospital laboratories, medical records departments, and infection control practitioners, and by reviewing databases of state-reportable conditions.

Once a person who met the surveillance case definition was identified, his or her hospital medical chart was abstracted and a standardized data collection instrument was completed. The instrument included a question about employment in selected health care occupations and an open text field to record other occupational information.

NIOSH received a deidentified dataset of 4,511 hospitalized cases of influenza from the 2009–10 influenza season from the CDC Influenza EIP program, as of May 18, 2010. One hundred forty-eight (3.3.%) case-patients were excluded from analyses because of incomplete data collection. An additional 11 (0.2%) case-patients were excluded because they were hospitalized outside the EIP catchment area, although they resided in a catchment area. We classified case-patients by work status (worker, nonworker, or unknown). Nonworkers included students, homemakers, retired persons, disabled persons, and nonworkers not elsewhere classified. Nonworkers not elsewhere classified included occupation text entries of none, not employed, unemployed, incarcerated, and homeless. All nursing home residents, regardless of occupational information recorded, were grouped with disabled persons because of their similar high prevalence of underlying medical conditions. If the text field for occupation was blank or indicated that the work status of the case-patient was unknown, we classified the work status as unknown. Trained NIOSH coders used responses to the health care worker question and occupational text entries to assign 2-digit codes for industry sector from the North American Industrial Classification System (*5*) and 2-digit codes for occupational group from the Standard Occupation Classification system (*6*) to the workers.

The EIP data collection instrument also included information about several underlying conditions associated with increased risk for influenza (asthma, cystic fibrosis, other chronic lung disease, chronic cardiovascular disease, renal disease, chronic metabolic disease [including diabetes], hemoglobinopathy, neuromuscular disorder, diagnosis of cancer [excluding nonmelanoma skin cancer] in the past 12 months, immunosuppressive condition, seizure disorder, Guillain-Barré syndrome, lymphoma or leukemia, cognitive dysfunction, pregnancy, and obesity). The EIP program does not collect any data on tobacco use, socioeconomic factors (e.g., income), or access to primary care among hospitalized influenza case-patients.

### National Health Interview Survey Reference Data for Employed US Population

Reference data for the employed US population was obtained from the 2010 National Health Interview Survey (NHIS) public use dataset (www.cdc.gov/nchs/nhis/quest_data_related_1997_forward.htm). The NHIS is a cross-sectional in-person household survey conducted annually by the National Center for Health Statistics, CDC. Data are collected on the civilian noninstitutionalized population of the United States, and thus exclude persons in long-term care facilities (e.g., nursing homes), correctional facilities, active-duty Armed Forces personnel (although civilian family members are included), and US nationals living in foreign countries. The survey uses a multistage clustered sample design with oversampling of black, Hispanic, and Asian persons and produces nationally representative data on health insurance coverage, health care access and use, health status, health behavior, and other health-related topics.

### Data Analyses

The proportions of adults hospitalized for influenza (EIP cases) by employment status were compared with expected proportions in the US population according to the 2010 NHIS, by age group. Among employed adults hospitalized for influenza, the proportions employed in each industry sector were compared with proportions of the US population employed in each industry sector. Ratios >1.0 indicated overrepresentation of an industry sector in the EIP dataset compared with what would be expected if workers from all industry sectors had the same risk for hospitalization because of influenza, which would lead to equal distributions of industry sectors between the 2 datasets. Confidence intervals were calculated by using the χ^2^ statistic to approximate the Poisson distribution.

We also used data from the 2010 NHIS to estimate the proportion of US adults employed in each sector who reported >1 underlying medical conditions, were current smokers, had relatively low annual earnings (<$35,000), and had a usual place to go for health care. We included health conditions reported in the NHIS that most closely matched the underlying health conditions for which data were collected in the EIP influenza study (i.e., asthma, emphysema, chronic bronchitis, cardiovascular disease [hypertension, coronary heart disease, angina, history of myocardial infarction, other heart condition, and/or history of stroke], renal failure, diabetes, diagnosis of cancer [excluding nonmelanoma skin cancer] in the past 12 months, epilepsy, pregnancy, and obesity [body mass index >30]). These estimates provide some group-level background information about underlying characteristics of workers employed in each industry sector that might affect their risks of hospitalization because of influenza (*4**,**7**–**12*).

Data analyses were performed by using SAS version 9.2 for Windows (SAS Institute, Cary, NC, USA). To account for the complex sampling design of the NHIS, all analyses of NHIS data were completed by using SAS survey procedures appropriate for complex samples and sampling information included in the public use dataset. Estimates based on NHIS data with relative SE >30% are not presented because of low reliability/precision.

## Results

Data were available for 4,352 case-patients who had laboratory-confirmed influenza and who were hospitalized during September 1, 2009–April 24, 2010. Of these case-patients, 3,365 (77.3%) had adequate information recorded to classify them according to work status: 1,283 workers, 96 students, 86 homemakers, 472 retired persons, 535 nursing home residents and disabled persons, and 893 nonworkers not elsewhere classified.

Overall, workers represent a much lower proportion of EIP hospitalized influenza case-patients compared with their proportions in the general US population according to NHIS data for every age group (EIP:NHIS ratio range 0.61–0.66) (Table 1). A total of 1,070 (83.4%) current workers were assigned 2-digit North American Industrial Classification System industry codes. Industry sectors with overrepresentation among hospitalized influenza case-patients (EIP data) compared with the 2010 NHIS reference population data were transportation and warehousing (ratio 1.53, 95% CI 1.18–1.94), administrative and support and waste management and remediation services (ratio 1.51, 95% CI 1.18–1.91), health care (ratio 1.47, 95% CI 1.26–1.70), and accommodation and food services (ratio 1.35, 95% CI 1.10–1.65) (Table 2).

**Table 1:** Employment status among US adults hospitalized with influenza September 1, 2009–April 24, 2010 by age group, compared with data from the 2010 NHIS*

Status	No. (%) cases†	Estimated US populations in thousands (weighted %) from NHIS†	Ratio (95% CI)
Age 18–49 y			
Employed	825 (45.2)	94,862 (71.3)	0.63 (0.59–0.68)
Not employed	999 (54.8)	38,170 (28.7)	1.91 (1.79–2.03)
Employment status unknown	586 (24.3)	36 (0)	NA
Age 50–64 y			
Employed	409 (41.2)	36,045 (62.4)	0.66 (0.60–0.73)
Not employed	583 (58.8)	21,675 (37.6)	1.56 (1.44–1.70)
Employment status unknown	300 (23.2)	24 (0)	NA
Age >65 y			
Employed	49 (8.9)	5,697 (14.7)	0.61 (0.45–0.80)
Not employed	500 (91.1)	32,980 (85.3)	1.07 (0.98–1.17)
Employment status unknown	101 (15.5)	15 (0)	NA

*NHIS, National Health Interview Survey; NA, not applicable. †Percentage among case-patients/respondents with known work status and among all case-patients/respondents with unknown work status.

**Table 2:** Hospitalized influenza case-patients by industry sector per EIP (September 1, 2009–April 24, 2010), ratios compared with distribution of employed US adults per NHIS, and characteristics of employed US adults by industry sector per NHIS*

Industry sector of employment (NAICS code)	% Employed hospitalized influenza case-patients, EIP	Weighted % employed adults, NHIS†	Ratio (95% CI)‡	% Case-patients with underlying condition, EIP§	% Adults, NHIS†			
					With underlying condition¶	Who smoke	Annual earnings <$35,00	With usual place for health care
Transportation and warehousing (48, 49)	6.26	4.10	1.53 (1.18–1.94)	76.12	54.07	20.79	48.23	78.36
Administrative and support and waste management and remediation (56)	6.64	4.38	1.51 (1.18–1.91)	73.24	49.82	25.34	80.74	67.69
Health care (62, except for 624)	16.17	11.01	1.47 (1.26–1.70)	81.40	52.46	16.24	59.12	87.72
Accommodation and food (72)	9.07	6.70	1.35 (1.10–1.65)	73.20	39.32	30.88	92.27	63.92
Other (81)	6.26	5.15	1.22 (0.94–1.54)	75.00	44.88	17.37	78.74	77.23
Social assistance (624)	3.18	2.70	1.18 (0.82–1.64)	82.35	60.66	14.32	81.83	89.66
Information ( 51)	2.90	2.54	1.14 (0.78–1.62)	87.10	51.19	15.54	44.20	84.80
Retail trade (44, 45)	12.43	11.03	1.13 (0.94–1.34)	85.61	46.35	22.64	80.93	75.84
Finance and insurance (52)	4.95	4.41	1.12 (0.84–1.47)	69.81	48.59	14.60	41.36	87.91
Education (61)	9.63	10.25	0.94 (0.77–1.14)	89.32	50.00	8.40	53.79	91.72
Professional, scientific, and technical (54)	6.26	6.84	0.92 (0.71–1.16)	71.64	41.06	13.35	28.65	85.08
Arts, entertainment, and recreation (71)	1.78	2.04	0.87 (0.52–1.36)	78.95	39.32	21.40	75.67	80.40
Construction (23)	5.05	6.61	0.76 (0.57–1.00)	57.41	46.50	29.09	63.51	70.01
Real estate and rental and leasing (53)	1.21	1.92	0.63 (0.34–1.08)	76.92	45.90	23.33	63.24	78.29
Public administration (92)	3.18	5.44	0.58 (0.40–0.82)	82.35	57.93	14.64	34.99	92.16
Manufacturing (31–33)	4.30	9.56	0.45 (0.33–0.60)	71.74	52.02	21.75	49.79	81.93
Utilities (22)	–	1.00	–	–	53.67	18.20	24.09	88.59
Agriculture, forestry, fishing, and hunting (11)	–	1.18	–	–	44.75	19.36	82.97	71.40
Wholesale trade (42)	–	2.58	–	–	49.76	23.71	48.68	82.70
Mining (21)	–	0.50	–	–	44.93	27.03	35.78	78.99
All employed	38.10	59.50	0.64 (0.59–0.70)	77.05	48.53	19.17	60.39	81.09
All nonemployed	61.90	40.50	1.53 (1.42–1.65)	87.56	65.20	19.62	96.77	84.52

*EIP, Emerging Infections Program; NHIS, National Health Interview Survey; NAICS, North American Industry Classification System; –, <5 cases. †Weighted estimates based on 2010 NHIS public use dataset (www.cdc.gov/nchs/nhis/quest_data_related_1997_forward.htm). ‡Proportion among EIP cases:proportion of employed adults, per NHIS. §Include asthma, cystic fibrosis, other chronic lung disease, chronic cardiovascular disease, renal disease, chronic metabolic disease (including diabetes), hemoglobinopathy, neuromuscular disorder, cancer (excluding nonmelanoma skin cancer) in past 12 mo, immunosuppressive condition, seizure disorder, Guillain-Barré syndrome, lymphoma or leukemia, cognitive dysfunction, pregnancy, and obesity. ¶Include asthma, emphysema, chronic bronchitis, cardiovascular disease (hypertension, coronary heart disease, angina, myocardial infarction, other heart condition, or stroke), renal failure, diabetes, cancer (excluding nonmelanoma skin cancer) in past 12 mo, epilepsy, pregnancy, and obesity (body mass index >30).

In general, industry sectors with the highest prevalence of underlying chronic medical conditions (per NHIS, e.g., social assistance, public administration) were not overrepresented in the EIP database (Table 2). For every industry sector, the proportion of workers with underlying medical conditions among hospitalized influenza (EIP) case-patients was higher than the proportion of workers in the general population (NHIS) with underlying medical conditions (Table 2). These ratios varied by industry sector, but neither the industry sector with the highest proportion of workers with underlying medical conditions among hospitalized influenza case-patients (education services) nor the industry sector with the highest ratio of case-patients with underlying conditions compared with workers in the general population with underlying conditions (arts, entertainment, and recreation) were overrepresented in the EIP database (Table 2).

According to the 2010 NHIS reference population data, some industry sectors overrepresented in the EIP database had a higher prevalence of demographic characteristics that might place them at increased risk for influenza-associated hospitalization. For example, the accommodation and food services industry sector had the highest prevalence of current smokers (30.9%) and workers with relatively low earnings (<$35,000 per year) (92.3%) (Table 2). With regards to health care access, the industry sectors with the lowest proportion of workers who report having a usual place to go for health care (other than an emergency department) include accommodation and food services (63.9%) and administrative and support and waste management and remediation services (67.7%) (Table 2).

## Discussion

Pandemic (H1N1) 2009 virus, which emerged in April 2009, resulted in substantial illness and deaths among working-aged adults. As reported, cumulative rates of laboratory-confirmed, influenza-associated hospitalizations in EIP sites were 2.4/10,000 population for all persons 18–49 years of age during August 30, 2009–March 27, 2010, which was ≈6× higher than the influenza-associated hospitalization rate for this age group in 2008–09, when seasonal influenza (H1N1) virus was the predominant strain (*13*).

We found that workers made up a much lower proportion of EIP hospitalized influenza case-patients than the general US population. This finding was not unexpected because of favorable underlying characteristics of workers compared with nonworkers, such as younger age and lower prevalence of diagnosed underlying medical conditions, which make workers less likely to be hospitalized for influenza. Furthermore, among workers, certain industry sectors are overrepresented in the EIP dataset than what would be expected if workers from all industry sectors had the same risk for hospitalization for influenza, suggesting that the risk for severe influenza varied among different groups of workers during the 2009–10 influenza season.

To date, most evaluations of work-related risk for influenza have focused on health care workers (*14**–**17*). There is some evidence that household exposures are more predictive of influenza infection among these workers than occupational exposures (*16*), but there is also evidence that occupational acquisition occurs (*14**,**17*). We found that persons working in the health care industry were overrepresented among hospitalized persons with influenza compared with what would be expected if workers from all industry sectors had the same risk for hospitalization because of influenza. However, health care workers were not the only worker group overrepresented.

Although the ratios were only modestly increased, these results suggest that groups of workers other than those employed in health care may also be at increased risk for influenza severe enough to result in hospitalization. Overrepresentation of an industry sector in the EIP dataset may be related to demographic and underlying health characteristics of the sector’s work force that put them at increased risk for acquiring influenza and for being hospitalized with influenza, but it may also partially reflect occupational risk factors for influenza (e.g., exposure to ill members of the public).

Because the EIP data only include cases, it is difficult to assess the potential reasons for overrepresentation of certain industry sectors, but general population estimates of potential contributing factors based on 2010 NHIS data provide some clues. We examined 4 factors for which data or expert consensus suggests associations with hospitalization (or severe outcomes in general) caused by influenza: underlying medical conditions (*4**,**7**,**8*), current smoking behavior (*8**,**9*), low income (*10**–**12*), and timely access to primary care (*8**,**10*), as measured by reporting a usual place to go for health care.

For example, the most highly overrepresented groups of workers among EIP cases, transportation and warehousing and administrative and support and waste management and remediation services, also had the highest prevalence of some unfavorable demographic characteristics, which might place them at increased risk for influenza-associated hospitalization. Workers in the accommodation and food services sector were also overrepresented among EIP cases. It seems logical that these workers may be at increased occupational risk for acquiring influenza because of their high level of interaction with the general public, but NHIS data also suggest some demographic factors that might increase their risk for hospitalization because of severe influenza (e.g., low earnings, smoking, lack of access to medical care).

On the other hand, variation in underlying health status, socioeconomic status, and access to health care by industry group does not appear to explain all of our findings regarding overrepresentation of groups among hospitalized influenza case-patients compared with the general working population. Health care workers are overrepresented among EIP cases despite being relatively healthy and having relatively high earnings and access to health care. Construction workers are underrepresented among EIP cases despite having relatively low earnings and access to health care. These workers in construction might have a relatively low risk for acquiring influenza because of low interaction with the public.

We also found some industry sectors in which we would expect a relatively high level of interaction with the public (e.g., public administration, education) that were not overrepresented among EIP cases. Even if these workers have an increased risk for acquiring influenza from the public, they might have a low risk for progressing to severe influenza requiring hospitalization because of their relatively high earnings and access to health care.

This study has several limitations in addition to the major limitation of relying on a secondary data source (NHIS) for information on the characteristics of workers by industry sector. No useful information on work status was available for 22.7% of EIP hospitalized influenza case-patients. This fact likely reflects the inconsistency of occupational data available in typical hospital records. There is the potential for misclassification of work status and, among workers, misclassification of industry sector because of inconsistency in narrative data recorded for occupation. Although we called the variable we collected occupation, there were more entries that reflected codable industry sectors than reflected codable occupational groups. Thus, we only reported results by industry. Furthermore, in most cases, the available information only enabled industry to be coded at a broad, nonspecific level. For example, it was impossible to distinguish whether many of the health care workers worked in inpatient or outpatient settings.

Our study examined systematically collected influenza surveillance data according to occupational variables. Benefits of using data from the EIP program include laboratory confirmation of influenza and representation of a large population from geographically diverse areas in the United States. Although we were able to identify specific groups of workers that were most heavily affected by severe influenza during the 2009–10 influenza season, more research is needed to understand the reasons for the increased incidence of severe influenza among specific groups of workers. Concurrently, any interventions that focus on these groups of workers should be evaluated for effectiveness and efficiency.

## References

[1] Occupational Safety and Health Administration, US Department of Labor. Guidance on preparing workplaces for an influenza pandemic. OSHA 3327–05R, 2009 [cited 2010 Jul 8]. http://www.osha.gov/Publications/OSHA3327pandemic.pdf

[2] Centers for Disease Control and Prevention. Guidance for businesses and employers to plan and respond to the 2009–2010 influenza season. Updated February 22, 2010 [cited 2010 Jul 8]. http://www.cdc.gov/h1n1flu/business/guidance/#top

[3] Suarthana E, McFadden JD, Laney AS, Kreiss K, Anderson HA, Hunt DC, Occupational distribution of persons with confirmed 2009 H1N1 influenza. J Occup Environ Med. 2010;52:1212–6. 10.1097/JOM.0b013e3181fd32e421124240

[4] Dao CN, Kamimoto L, Nowell M, Reingold A, Gershman K, Meek J, Adult hospitalizations for laboratory-positive influenza during the 2005–2006 through 2007–2008 seasons in the United States. J Infect Dis. 2010;202:881–8. 10.1086/65590420677944

[5] Office of Management and Budget. North American industry classification system (NAICS). United States 2007 [cited 2012 Jan 6]. http://www.census.gov/naics

[6] Office of Management and Budget. Standard occupational classification manual (SOC). United States 2000 [cited 2012 Jan 6]. http://stats.bls.gov/soc/soc_home.htm

[7] Jain S, Kamimoto L, Bramley AM, Schmitz AM, Benoit SR, Louie J, Hospitalized patients with 2009 H1N1 influenza in the United States, April—June 2009. N Engl J Med. 2009;361:1935–44. 10.1056/NEJMoa090669519815859

[8] Gilca R, De Serres G, Boulianne N, Ouhoummane N, Papenburg J, Douville-Fradet M, Risk factors for hospitalization and severe outcomes of 2009 pandemic H1N1 influenza in Quebec, Canada. Influenza Other Respi Viruses. 2011;5:247–55. 10.1111/j.1750-2659.2011.00204.x21651735PMC4634547

[9] Huttunen R, Heikkinen T, Syrjänen J. Smoking and the outcome of infection. J Intern Med. 2011;269:258–69. 10.1111/j.1365-2796.2010.02332.x21175903

[10] Bouye K, Truman BI, Hutchins S, Richard R, Brown C, Guillory JA, Pandemic influenza preparedness and response among public-housing residents, single-parent families, and low-income populations. Am J Public Health. 2009;99:S287–93. 10.2105/AJPH.2009.16513419797740PMC4504369

[11] Thompson DL, Jungk J, Hancock E, Smelser C, Landen M, Nichols M, Risk factors for 2009 pandemic influenza A (H1N1)–related hospitalization and death among racial/ethnic groups in New Mexico. Am J Public Health. 2011;101:1776–84.2177849510.2105/AJPH.2011.300223PMC3154223

[12] Yousey-Hindes KM, Hadler JL. Neighborhood socioeconomic status and influenza hospitalizations among children: New Haven County, Connecticut, 2003–2010. Am J Public Health. 2011;101:1785–9.2177849810.2105/AJPH.2011.300224PMC3154248

[13] Centers for Disease Control and Prevention. Update: influenza—United States, August 30, 2009–March 27, 2010, and composition of the 2010–11 influenza vaccine. MMWR Morb Mortal Wkly Rep. 2010;59:423–30.20395936

[14] Centers for Disease Control and Prevention. Novel influenza A (H1N1) virus infections among healthcare personnel—United States, April–May 2009. MMWR Morb Mortal Wkly Rep. 2009;58:641–5.19543199

[15] Santos CD, Bristow RB, Vorenkamp JV. Which healthcare workers were most affected during the spring 2009 H1N1 pandemic? Disaster Med Public Health Prep. 2010;4:47–54.2038919510.1017/s193578930000241x

[16] Williams CJ, Schweiger B, Diner G, Haaman F, Krause G, Nienhaus A, Seasonal influenza risk in hospital healthcare workers is more strongly associated with household than occupational exposures: results from a prospective cohort study in Berlin, Germany, 2006/07. BMC Infect Dis. 2010;10:8. 10.1186/1471-2334-10-820067628PMC2836320

[17] Wise ME, De Perio M, Halpin J, Jhung M, Magill S, Black SR, Transmission of pandemic (H1N1) 2009 influenza to healthcare personnel in the United States. Clin Infect Dis. 2011;52:S198–204. 10.1093/cid/ciq03821342895

# Research article

**DOI:** 10.3201/eid1804.110337

## Medscape CME Quiz

## Earning CME Credit

To obtain credit, you should first read the journal article. After reading the article, you should be able to answer the following, related, multiple-choice questions. To complete the questions (with a minimum 70% passing score) and earn continuing medical education (CME) credit, please go to www.medscape.org/journal/eid. Credit cannot be obtained for tests completed on paper, although you may use the worksheet below to keep a record of your answers. You must be a registered user on Medscape.org. If you are not registered on Medscape.org, please click on the New Users: Free Registration link on the left hand side of the website to register. Only one answer is correct for each question. Once you successfully answer all post-test questions you will be able to view and/or print your certificate. For questions regarding the content of this activity, contact the accredited provider, CME@medscape.net. For technical assistance, contact CME@webmd.net. American Medical Association’s Physician’s Recognition Award (AMA PRA) credits are accepted in the US as evidence of participation in CME activities. For further information on this award, please refer to http://www.ama-assn.org/ama/pub/category/2922.html. The AMA has determined that physicians not licensed in the US who participate in this CME activity are eligible for *AMA PRA Category 1 Credits*™. Through agreements that the AMA has made with agencies in some countries, AMA PRA credit may be acceptable as evidence of participation in CME activities. If you are not licensed in the US, please complete the questions online, print the certificate and present it to your national medical association for review.

## Article Title: Influenza-associated Hospitalizations by Industry, 2009–10 Influenza Season, United States

## CME Questions

**1. Which of the following statements regarding employment and the risk for hospitalization for influenza in the current study is most accurate?**

A. There was a broad increase in the risk for hospitalization for influenza among unemployed vs. employed individuals

B. Employment was a risk factor for hospitalization for influenza only among older adults

C. Employment was a risk factor for hospitalization for influenza only among retail workers

D. There was no relationship between employment status and the risk for hospitalization for influenza

**2. All of the following industry sectors experienced overrepresentation of hospitalized influenza cases EXCEPT:**

A. Transportation and warehousing

B. Public administration

C. Healthcare

D. Accommodation and food service

**3. Which of the following statements regarding the interaction between underlying medical conditions and the risk for hospitalization for influenza in the current study is most accurate?**

A. Underlying medical conditions were associated with an increased risk for hospitalization in all industry sectors

B. Educational services carried the highest risk for hospitalization due to its high rate of underlying medical conditions

C. Arts and entertainment carried the highest risk for hospitalization due to its high rate of underlying medical conditions

D. Underlying medical conditions were a risk factor for hospitalization only among unemployed individuals

**4. What other factors might have most influenced the rate of hospitalization for influenza in the current study?**

A. Access to healthcare

B. Female sex

C. Location of employment

D. Race other than white or black

## Activity Evaluation

**Table:** 

1. The activity supported the learning objectives.				
Strongly Disagree				Strongly Agree
1	2	3	4	5
2. The material was organized clearly for learning to occur.				
Strongly Disagree				Strongly Agree
1	2	3	4	5
3. The content learned from this activity will impact my practice.				
Strongly Disagree				Strongly Agree
1	2	3	4	5
4. The activity was presented objectively and free of commercial bias.				
Strongly Disagree				Strongly Agree
1	2	3	4	5
